# Hydrogen Sulfide Protects HUVECs against Hydrogen Peroxide Induced Mitochondrial Dysfunction and Oxidative Stress

**DOI:** 10.1371/journal.pone.0053147

**Published:** 2013-02-05

**Authors:** Ya-Dan Wen, Hong Wang, Sok-Hong Kho, Suguro Rinkiko, Xiong Sheng, Han-Ming Shen, Yi-Zhun Zhu

**Affiliations:** 1 Department of Pharmacology, School of Medicine, National University of Singapore, Singapore, Singapore; 2 Institute of Biomedicine, Jinan University, Guangzhou, China; 3 Saw Swee Hock School of Public Health, National University of Singapore, Singapore, Singapore; University of Valencia, Spain

## Abstract

**Background:**

Hydrogen sulfide (H_2_S) has been shown to have cytoprotective effects in models of hypertension, ischemia/reperfusion and Alzheimer's disease. However, little is known about its effects or mechanisms of action in atherosclerosis. Therefore, in the current study we evaluated the pharmacological effects of H_2_S on antioxidant defenses and mitochondria protection against hydrogen peroxide (H_2_O_2_) induced endothelial cells damage.

**Methodology and Principal Findings:**

H_2_S, at non-cytotoxic levels, exerts a concentration dependent protective effect in human umbilical vein endothelial cells (HUVECs) exposed to H_2_O_2_. Analysis of ATP synthesis, mitochondrial membrane potential (Δ*Ψ*m) and cytochrome c release from mitochondria indicated that mitochondrial function was preserved by pretreatment with H_2_S. In contrast, in H_2_O_2_ exposed endothelial cells mitochondria appeared swollen or ruptured. In additional experiments, H_2_S was also found to preserve the activities and protein expressions levels of the antioxidants enzymes, superoxide dismutase, catalase, glutathione peroxidase and glutathione-S-transferase in H_2_O_2_ exposed cells. ROS and lipid peroxidation, as assessed by measuring H_2_DCFDA, dihydroethidium (DHE), diphenyl-l-pyrenylphosphine (DPPP) and malonaldehyde (MDA) levels, were also inhibited by H_2_S treatment. Interestingly, in the current model, D, L-propargylglycine (PAG), a selective inhibitor of cystathionine γ-lyase (CSE), abolished the protective effects of H_2_S donors.

**Innovation:**

This study is the first to show that H_2_S can inhibit H_2_O_2_ mediated mitochondrial dysfunction in human endothelial cells by preserving antioxidant defences.

**Significance:**

H_2_S may protect against atherosclerosis by preventing H_2_O_2_ induced injury to endothelial cells. These effects appear to be mediated via the preservation of mitochondrial function and by reducing the deleterious effects of oxidative stress.

## Introduction

Atherosclerosis is a chronic inflammatory response to high serum cholesterol levels, leading to plaques formation and the hardening of arteries. Cumulatively these effects increase an individuals risk of stroke, myocardial infarction or additional systemic complications [1]. The progressive nature of this disease, that can remain undetected for many years, is induced and maintained by several mechanisms including oxidative stress, inflammation, cell adhesion and cellular proliferation [2]. These combined changes lead to endothelial dysfunction. Importantly, the endothelium functions as an impermeable barrier whose integrity plays important roles in prohibiting leukocyte adhesion, reducing inflammation and supporting the vasculature that release paracrine signaling peptides to regulate vascular tone during hemodynamic stresses and oxidative stress [3]. Current treatments to preserve the endothelium include the lowering of serum lipids using statins. However, the development of new pharmacological drugs affecting multiple targets like oxidative stress and mitochondria may afford better protection against atherosclerosis.

H_2_S has recently been identified as a novel gaseous signaling molecule synthesized by cystathionine-γ-lyase (CSE) in the cardiovascular system. H_2_S has cardioprotective effects, as determined in a number of *in vitro* and *in vivo* animal models. H_2_S lowers blood pressure in spontaneous hypertensive rats (SHRs) [4] and in CSE gene knockout mice (SMCs-KO) [5], inhibits vascular remodeling induced by hypertension [6], inhibits oxidized LDL formation *in vitro* [7], and suppresses the toxicity of reactive oxygen species (ROS) [8]. H_2_S hypotensive, antioxidative and anti-inflammatory effects are well documented, however, its influences on atherosclerosis, especially upon endothelial cells, is far from clear.

Oxidative stress is a potent pathogenic mechanism in atherosclerosis [9] by promoting cellular injury, mitochondria dysfunction and apoptosis [10]. Mitochondria play crucial roles in the regulation of the cell cycle, cell growth and cell death [11]. To date, few studies have been conducted to determine the regulatory role that H_2_S may play on preserving tissue antioxidant defensives and mitochondrial function in endothelial cells.

Therefore, in the current studies we have examined the protective effects of H_2_S in endothelial cells against H_2_O_2_ induced cellular stress. Our major focus is to determine whether H_2_S, i) executes it protective effects through CSE/H_2_S pathway, ii) preserves mitochondrial functions and ultrastructure, iii) promotes the induction of cytoprotective antioxidant enzymes that can detoxify toxic free radicals. The current findings provide further evidences for a functional role of H_2_S in endothelial cells in relation to the prevention of atherosclerosis.

## Results

### NaHS is non-toxic to HUVECs

**Figure 1:**
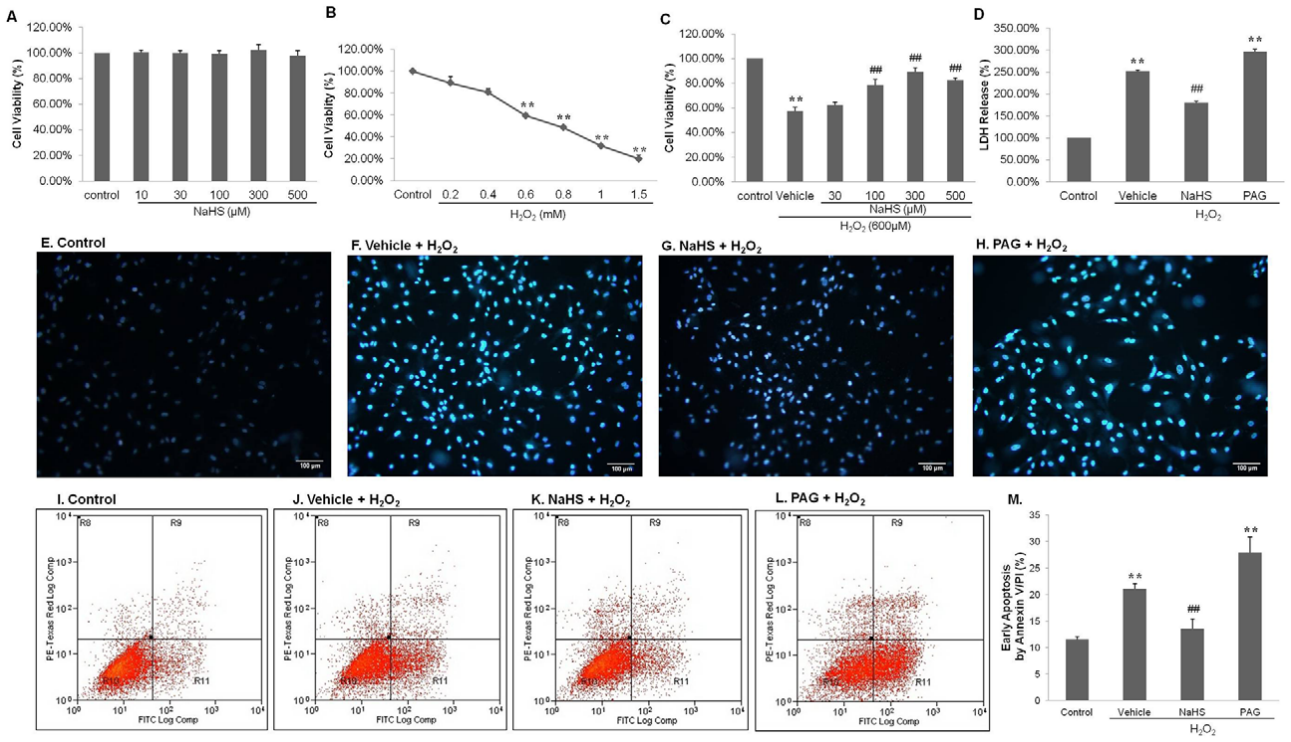
Cell viability and death assay of HUVECs subjected to different concentrations of NaHS with or without H_2_O_2_. (**A**)–(**C**) MTT assay. (**A**) HUVECs were treated with 10–500 μM NaHS for 6 hours. (**B**) HUVECs were treated with 0.2–1.5 mM H_2_O_2_ for 4 hours. (**C**) HUVECs were pretreated with vehicle or 30–500 μM NaHS for 6 hours, followed by exposure to 600 μM H_2_O_2_ for another 4 hours. (**D**) LDH Release. HUVECs were pretreated with vehicle or 300 μM NaHS and 10 mM PAG for 6 hours, followed by exposure to 600 μM H_2_O_2_ for another 4 hours. Cell viability in each treatment group is expressed as a percentage of control. (**E**)–(**H**) Hoechst staining. HUVECs were pretreated with vehicle, 300 μM NaHS or 10 mM PAG for 6 hours, followed by exposure to 600 μM H_2_O_2_ for another 4 hours. Cells were observed under ×200 microscopy. Scale bar is shown at 100 μm. (**I**)–(**M**) Annexin V/PI staining detected by flow cytometry. HUVECs were pretreated with vehicle or 300 μM NaHS and 10 mM PAG for 6 hours, followed by exposure to 600 μM H_2_O_2_ for another 4 hours. The data shown are mean ± SEM (n = 9). ** *p*<0.01 vs control. ## *p*<0.01 vs vehicle + H_2_O_2_.

Sodium hydrosulfide (NaHS) at concentrations of 10, 30, 100, 300 and 500 μM was found to be non-toxic to endothelial cells, as determined by the MTT viability assay (Fig 1 A, n = 9).

### Protective effects of exogenous H_2_S on H_2_O_2_ induced cell death

The dose response of H_2_O_2_ treatments on HUVECs cell viability is shown in Fig. 1 B (n = 9). After incubating with H_2_O_2_ for 4 hours, cell viabilities were significantly decreased in 0.6–1.5 mM H_2_O_2_ treatment groups (*P*<0.01). The less toxic concentration, 0.6 mM of H_2_O_2_ was chosen for later studies. Another MTT assay (Fig. 1C) showed cell viability fell to 57.71±2.96% when exposed to H_2_O_2_ (600 µM) for 4 h (*P*<0.01, vs. control). A dose response was observed in cell viabilities 62.38±2.12%, 78.74±4.23%, 89.26±3.45% and 82.41±1.78% for 30, 100, 300 and 500 µM NaHS, respectively. The differences between the H_2_O_2_ group and NaHS (100, 300 and 500 µM) groups were statistically significant (*P*<0.01). The MTT results were further supported by lactate dehydrogenase (LDH) release assay (Fig. 1D). Compared with control, vehicle + H_2_O_2_ induced 252.23±1.79% LDH release, while NaHS pretreatment significantly decreased LDH release to 180.63±3.13% and PAG increased to 297.26±5.28% (*P*<0.01). Pretreatment with different concentrations of NaHS could reverse H_2_O_2_-induced cell death dramatically, showing the ability of H_2_S to reduce H_2_O_2_ cytotoxicity. Similar results were obtained using Hoechst staining (Fig. 1 E–H), in which apoptotic nuclei were brighter. There were fewer apoptotic cells in the NaHS pre-treated groups than in the H_2_O_2_ group, while much more apoptotic cells in the PAG pre-treated groups were observed than in control group. Fig. 1I–M showed the protective effects of H_2_S on cells in the stage of early apoptosis induced by H_2_O_2_. The percentage of cells stained by Annexin V/PI which indicated early apoptosis, was 11.5±0.53% in control, and significantly increased to 21.33±0.89% in vehicle, decreased to 13.59±1.77% in NaHS and highly rocketed to 27.81±3% in PAG (*P*<0.01). H_2_S can protect endothelial cells against H_2_O_2_-induced apoptosis.

### CSE protein and mRNA expression, CSE activity and H_2_S concentration after H_2_O_2_-induced injury

**Figure 2:**
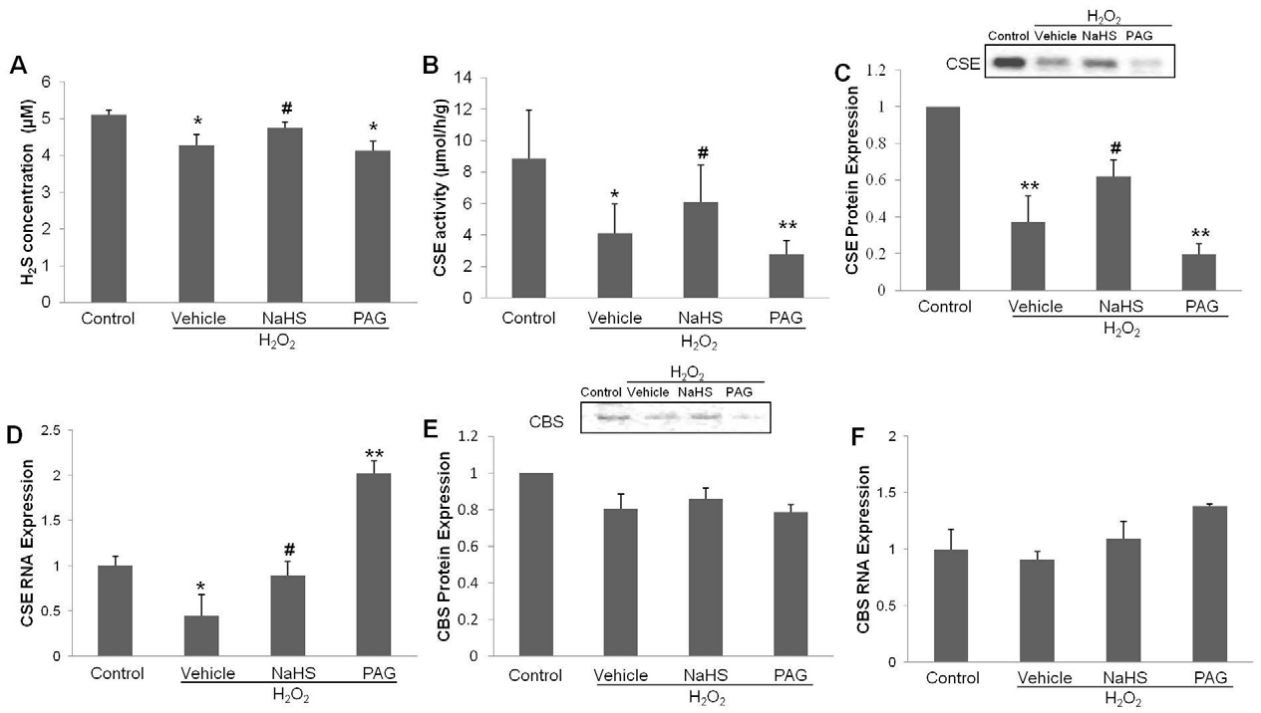
Effects of NaHS on H_2_S levels and H_2_S synthesizing enzyme activities. (**A**) The changes of H_2_S levels in medium for each treatment group (expressed in μM). (**B**) CSE activities in HUVECs lysate of each group, presented as μmol/h/g. (**C**) CSE protein expressions levels as determined using western blot analysis. (**D**) CSE mRNA expression levels as determined by real-time PCR. (**E**) CBS protein expressions levels and (**F**) The CBS mRNA expression tested by real-time PCR. The values in (C)–(F) were normalized against the control values. The data shown are mean ± SEM (n = 6). * *p*<0.05, ** *p*<0.01 vs control. # *p*<0.05 vs vehicle + H_2_O_2_ group.

H_2_O_2_ treatment was found to decrease of H_2_S concentration in the medium, Fig. 2 A (n = 6) (*P*<0.05, vs. control). In contrast, the H_2_S donor NaHS elevated H_2_S concentrations in the media (*P*<0.05, vs. vehicle + H_2_O_2_). These effects were significantly reduced by PAG treatment (*P*<0.05, vs. control). Similarly, cellular CSE activities were analyzed in the cell lysates from all treatment groups, as shown in Fig. 2 B (n = 6). CSE activity in the control group was 28.73±0.69 (μmol/h/g) with this been decreased to 15.02±0.91 (μmol/h/g) in the H_2_O_2_ group (*P*<0.05, vs. control). NaHS was found to preserve CSE activity in cells when exposed to H_2_O_2_ (21.07±0.52 μmol/h/g; *P*<0.05, vs. vehicle + H_2_O_2_), while PAG reduced CSE activity levels to 13.02±0.97 (μmol/h/g) (*P*<0.01, vs. control). Using western blot and PCR analysis we also determined the relative protein and mRNA levels of CSE in HUVECs (Fig. 2 C, D). In the H_2_O_2_ treatment groups CSE protein and mRNA levels were reduced while in the NaHS pretreatment groups CSE protein and mRNA levels were increased and in the PAG pretreatment groups CSE protein levels were decreased and CSE mRNA levels were increased (*P*<0.05). Interestingly, CBS protein and mRNA levels, an additional H_2_S synthesizing enzyme, remained unchanged in all treatments groups as shown in Fig. 2 E–F. Taken together, these results indicate that the cardioprotective effects by H_2_S might be though the CSE/H_2_S pathways.

### Effects of exogenous H_2_S on mitochondrial ATP synthesis

**Figure 3:**
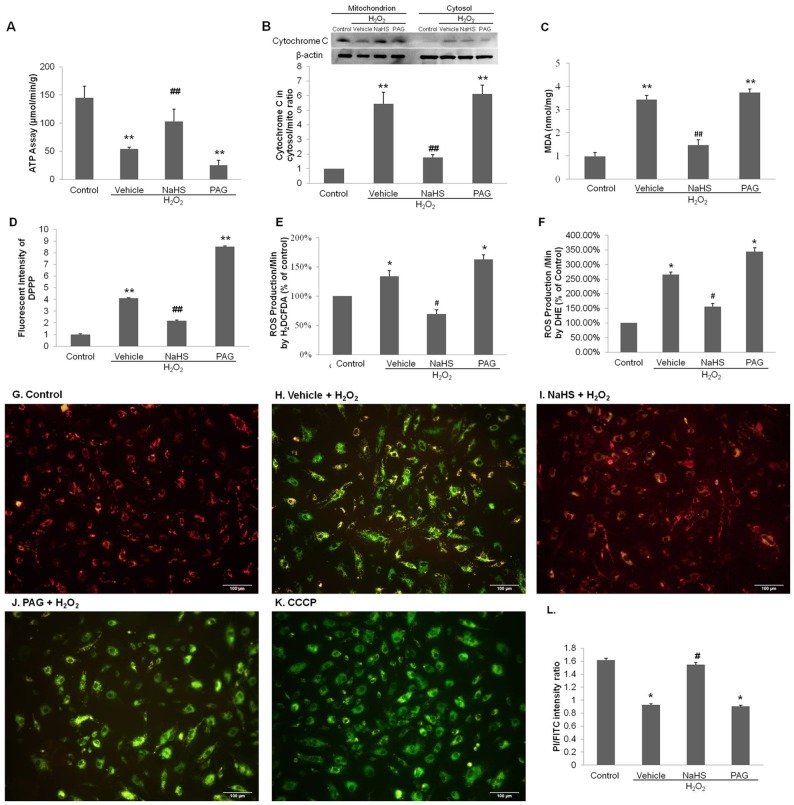
Effects of NaHS on mitochondrial function. (**A**) ATP synthesis. After pretreatment with vehicle, 300 μM NaHS or 10 mM PAG for 6 hours and followed by exposure to 600 μM H_2_O_2_ for another 4 hours, HUVECs were harvested to collect mitochondria. The rate of ATP synthesis was expressed by µmol ATP/min/g of mitochondrial protein. (**B**) Release of cytochrome c from mitochondria. After treatments as previous description, HUVECs were harvested to collect mitochondria and cytosol. The protein expression was tested by western blot. The bar chart showed the ratio of cytochrome c in cytosol to that in mitochondria, indicating the intensity of release of cytochrome c. (**C**) MDA changes in HUVECs mediated by H_2_O_2_. The data are expressed at nmol/mg. (**D**) Fluorescent intensity of DPPP in HUVECs mediated by H_2_O_2_. (**E**) ROS production was stained by 10 μM H_2_DCFDA for 20 min, whose oxidation product (DCF) fluorescence indicated ROS formation. (**F**) ROS production was stained by 5 μM DHE for 30 min, which fluorescence indicated ROS formation. The absorbance values in (D)–(F) of HUVECs were normalized against the values for normal controls and expressed as a percentage of control. (**G**)–(**L**) JC-1 staining. Red fluorescence represents the mitochondrial aggregate form of JC-1, indicating intact mitochondrial membrane potential. Green fluorescence represents the monomeric form of JC-1, indicating dissipation of Δ*Ψ*_m_. (G)–(J) HUVECs were stained with JC-1. (K) CCCP was the positive control. Cells were observed under ×200 microscopy. Scale bar is shown at 100 μm. (L) Ratio of red to green fluorescence, indicating ratio of JC-1 polymer/monomer. The data shown are mean ± SEM (n = 6). * *p*<0.05, ** *p*<0.01vs control. # *p*<0.05, ## *p*<0.01 vs vehicle + H_2_O_2_ group.

In aerobic eukaryote cells the major site of adenosine triphosphate (ATP) production occurs in mitochondria. As shown in Fig. 3A (n = 6) cellular ATP levels responded to H_2_O_2_, the H_2_S donor (NaHS) and inhibitor (PAG). The ATP content of the control cells was 144.66±21.13 (µmol/min/g). After incubation with H_2_O_2_, the rate of ATP production by mitochondria was greatly decreased to 54.16±2.79 (µmol/min/g) (P<0.01, vs. control). Meanwhile, ATP production in the pretreated NaHS group significantly increased to 102.87±22.34 (µmol/min/g) (*P*<0.01, vs. vehicle + H_2_O_2_). A significantly decreased to 25.47±8.90 (µmol/min/g) in the pretreated PAG group was also noted (*P*<0.01, vs. control). These results indicated that H_2_S could attenuate H_2_O_2_ induced inhibition of ATP synthesis.

### Effects of exogenous H_2_S on mitochondrial membrane permeability

JC-1 aggregates in healthy mitochondria and has a red fluorescence (Fig. 3G). Exposure of HUVECs to H_2_O_2_ resulted in an increase in green fluorescence, indicating a loss in mitochondrial membrane potential (Δ*Ψ*_m_) (Fig. 3H). Pretreatment of NaHS reduced the effects of H_2_O_2_ on mitochondrial membrane potential, indicating a protective effect of NaHS (Fig. 3I). Pretreatment with PAG also resulted in the dissipation of mitochondrial membrane potential (Fig. 3J). Carbonyl cyanide m-chlorophenylhydrazone (CCCP), the positive control, promoted mitochondrial inner membrane permeable leading to the dissipation of the proton gradient across the inner mitochondrial membrane (Fig. 3K). The ratio of red and green fluorescence was also used to demonstrate the toxicity of H_2_O_2_ treatment to mitochondria and the protective effect of NaHS (Fig. 3L). Moreover, the ratio of aggregated JC-1 and monomeric JC-1 were measured by flow cytometry. In control cells, JC-1 aggregated in mitochondria. In contrast, in H_2_O_2_-treated cells a lower ratio was observed (*P*<0.05, vs. control) because the monomeric form of JC-1 appeared in the cytosol indicating the dissipation of Δ*Ψ*_m_. Cells pre-treated with NaHS attenuated the dissipation of Δ*Ψ*_m_ (*P*<0.05, vs. vehicle + H_2_O_2_), while PAG further increased the loss of membrane potential (*P*<0.05, vs. control).

One possible mechanism by which oxidative stress may trigger cellular toxicity in HUVECs is the induction of the mitochondrial apoptotic pathway that is triggered through the release of cytochrome c into the cytosol. To verify this possibility, the protective effect of NaHS on H_2_O_2_-induced toxicity was measured by determining the release of cytochrome c from mitochondria using Western-Blot analysis (Fig. 3B) (n = 6). In the control group, the presence of relatively low levels of cytochrome c was released from the mitochondria to cytosol. After incubation with H_2_O_2_, the cytochrome c levels were significantly increased in the cytosol and decreased in mitochondria (*P*<0.01, vs. control). Pretreatment with NaHS inhibited the release of cytochrome c (*P*<0.01, vs. vehicle + H_2_O_2_), while pretreatment of PAG potentiated the release of cytochrome c to the cytosol (*P*<0.01, vs. control).

### Endothelial cell ultrastructure observation

**Figure 4:**
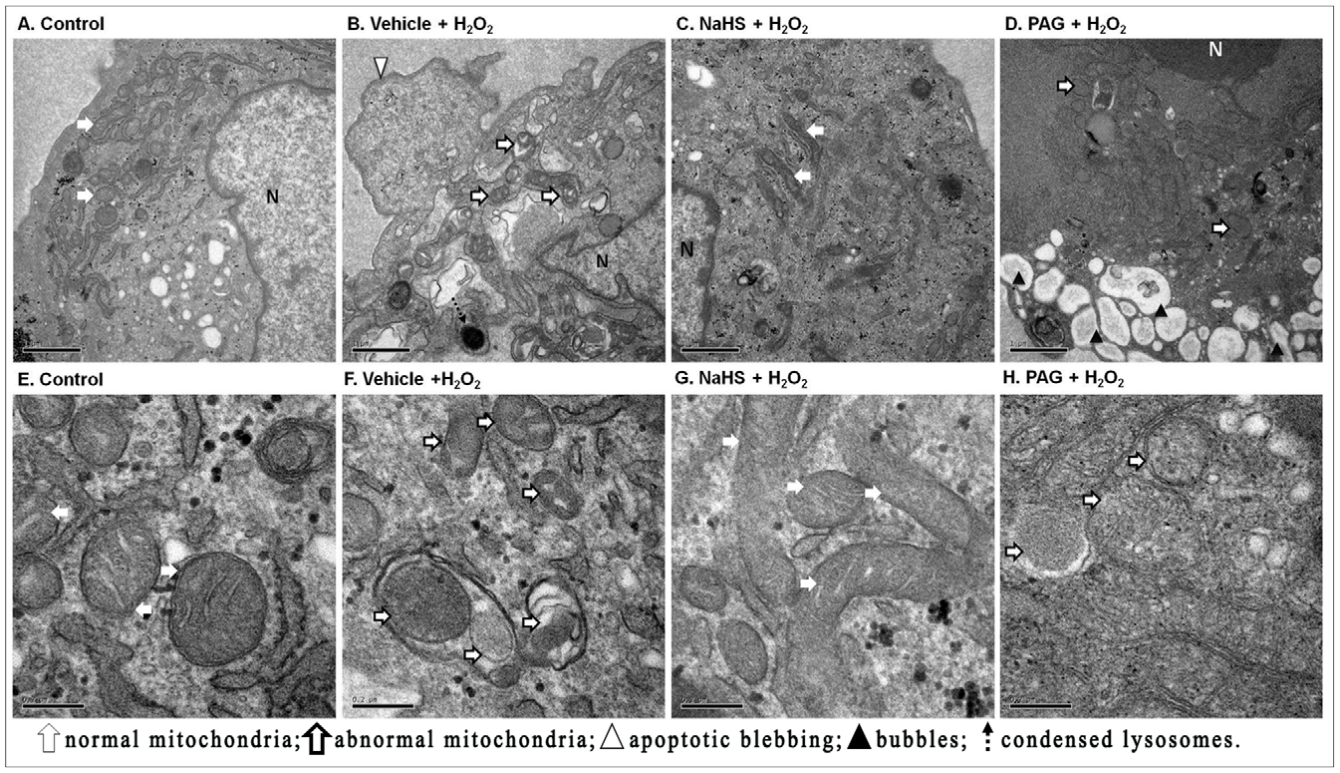
Ultrastructural changes in HUVECs induced by H_2_O_2_ using transmission electron microscopy. (**A**)–(**D**) showed HUVECs with legible nucleus. Scale bar is shown at 1 μm. (**E**)–(**H**) showed mitochondria. Scale bar is shown at 0.2 μm. (**A**) and (**E**) cell and mitochondria in the control group; (**B**) and (**F**) cell and mitochondria in vehicle + H_2_O_2_ group; (**C**) and (**G**) cell and mitochondria in NaHS + H_2_O_2_ group; (**D**) and (**H**) cell and mitochondria in PAG + H_2_O_2_ group.

Fig. 4 shows ultrastructural changes in HUVECs exposed to H_2_O_2_. In control cells the ultrastructure was normal with intact nuclei and healthy looking mitochondria, endoplasmic reticulum and lysosomes (Fig. 4 A, E). In contrast, H_2_O_2_-treated HUVECs displayed condensed chromatin, an irregular nuclear outline, dilated or fragmented endoplasmic reticulum, swollen, ruptured or engulfed mitochondria, and darkened and clumping lysosomes. In addition the cytoplasm had significant vacuolization and protrusions (Fig. 4 B, F). In the NaHS pretreated HUVECs, the cellular ultrastructure appeared similar to that of the control endothelial cells although some mitochondria had expanded cristas, and darkened lysosomes, vacuoles and engulfed organelles (Fig. 4 C, G). In the PAG pretreated cells a more severe ultrastructural change was observed including nuclear chromatin condensation, multiple cytoplasmic protrusions or blebs, and ruptured or fragmented organelles (Fig. 4 D, H). Essentially, the transmission electron microscopic observations indicated that exogenous H_2_S could preserve cellular ultrastructural changes induced by H_2_O_2_.

### Effects of exogenous H_2_S on MDA formation and ROS production

MDA is a product of lipid peroxidation. The data for the levels of cellular MDA are shown in Fig. 3C (n = 6). MDA levels were low in the normal control cells. Treatment with H_2_O_2_ significantly increased cellular MDA levels indicating the elevation of oxidative stress (*P*<0.01, vs. control). Pretreatment with NaHS reduced the formation of MDA induced by H_2_O_2_ (*P*<0.01, vs. vehicle + H_2_O_2_), while pretreated PAG reversed the inhibition caused by NaHS (*P*<0.01, vs. control). The study of DPPP staining was in line with MDA result, which showed the increase in fluorescent intensity of DPPP induced by H_2_O_2_ was reduced by NaHS and enhanced by PAG (*P*<0.01) (Fig. 3D).

Redox status was observed in H_2_DCFDA study (Fig. 3E) (n = 6). Administration of H_2_O_2_ induced an increase in the fluorescence intensity of H_2_DCFDA, as compared to the control group (*P*<0.05). Pre-incubation with NaHS inhibited the levels of ROS induced by H_2_O_2_ (*P*<0.05). However, pretreatment with PAG intensified the fluorescence intensity of H_2_DCFDA induced by H_2_O_2_ (*P*<0.05). DHE is another dye for ROS detection, especially superoxide. Similar results were found that cells stimulated by H_2_O_2_ expressed higher fluorescent intensity of DHE than that of control, which were suppressed by NaHS and strengthened by PAG (*P*<0.05) (n = 6) (Fig. 3F). Our results suggest that exogenous H_2_S can suppress the productions of toxic free radicals.

### Effects of exogenous H_2_S on antioxidants activities and antioxidants enzyme protein expressions

The activities of tissue antioxidant enzymes are shown in Table 1. When cells were stimulated with H_2_O_2_ a significant decreases in the activities of superoxide dismutase (SOD), catalase, glutathione Peroxidase (GPx) and glutathione S-transferase (GST) were observed (*P*<0.05, vs. control, n = 6). However, pretreatment with NaHS significantly elevated the activities of SOD, catalase, GST and GPx (*P*<0.05, vs. vehicle + H_2_O_2_), while PAG strongly reduced the activities of these antioxidative enzymes (*P*<0.01, vs. control). In our study, exogenous H_2_S was found to increase the activities of antioxidant enzymes as compared with H_2_O_2_-stimulated group.

**Table 1:** Antioxidant enzyme activities in each study groups.

	SOD (U/g )	Catalase(nmol/min/g)	GPx(nmol/min/g)	GST(nmol/min/g)
**Control**	4.32±0.84	12.39±1.75	68.85±5.63	45.66±4.8
**H_2_O_2_**	1.24±0.14**	7.35±1.08**	48.11±6.21**	27.41±4.0**
**NaHS**	3.98±0.53^##^	9.42±1.38^#^	55.39±13.67^#^	32.48±3.35^##^
**PAG**	0.82±0.11**	6.09±1.34**	42.85±6.23**	19.41±1.62**

The data shown are mean ± SEM (n = 6). ** *p*<0.01 vs control. # *p*<0.05, ## *p*<0.01 vs vehicle + H_2_O_2_ group.

**Figure 5:**
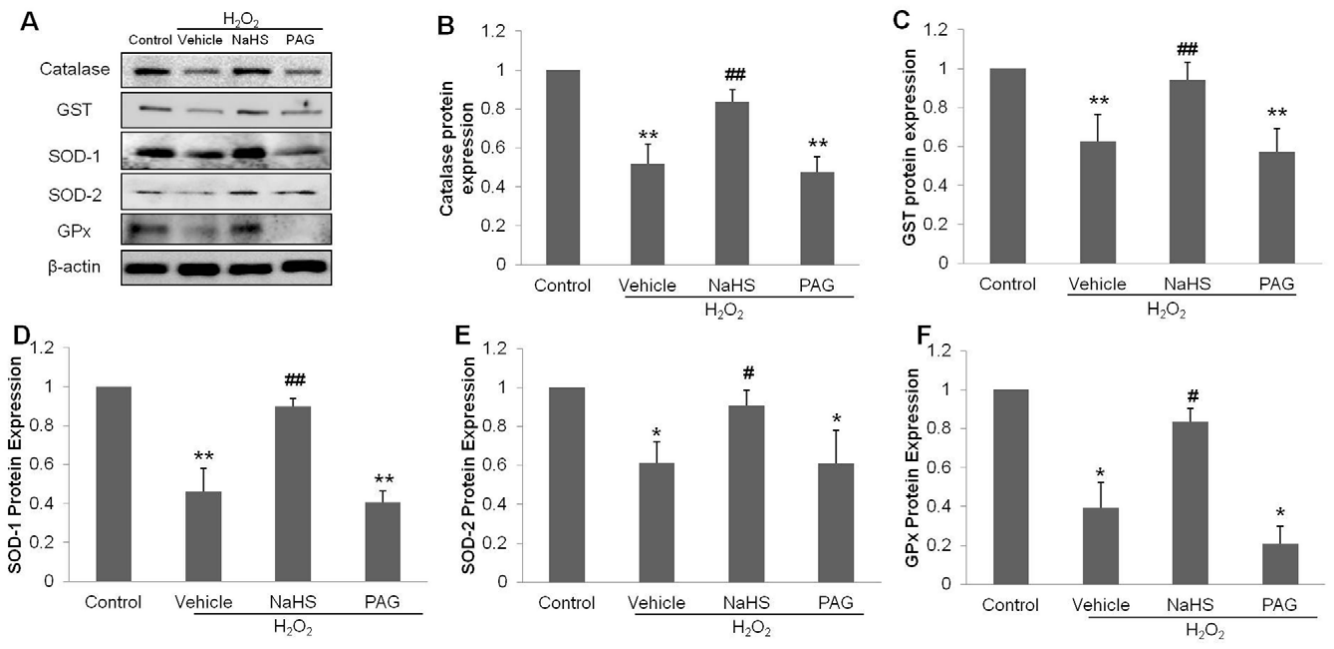
Effects of NaHS on protein expressions of antioxidant enzymes. (**A**) Western-blot analysis showing the intensities of Catalase, SOD-1, SOD-2, GST and GPx in each group, (**B**)–(**F**) bar charts indicating the different intensities of antioxidant proteins between groups. Values were normalized against the control values. The data shown are mean ± SEM (n = 6). * *p*<0.05, ** *p*<0.01 vs control. # *p*<0.05, ## *p*<0.01 vs vehicle + H_2_O_2_ group.

To support these findings, we examined the protein expression levels of SOD, catalase, GPx and GST in HUVECs, as shown in Fig. 5 A–F (n = 6). Protein expression levels were all decreased in the H_2_O_2_ and PAG groups as compared to the control group (*P*<0.05). In the NaHS group higher protein expressions levels of antioxidant enzymes was observed as compared to the H_2_O_2_ group (*P*<0.05). These results are in line with the corresponding enzymes activities in the above experiments. Combined these data indicates that H_2_S can enhance the antioxidative systems in cells under H_2_O_2_-stimulated stress and thus preserve cellular redox balance. These data correlating with the preservation of mitochondrial integrity, reduced oxidative stress levels and the maintenance of cell viability by H_2_S.

## Discussion

This study explored the cardiovascular protective effects by H_2_S on H_2_O_2_ induced injured in HUVECs as assessed by measurements of mitochondrial integrity and antioxidative systems. It is well known that oxidative stress is a potent atherogenic factor during the initiation of atherosclerotic lesions. Oxidized cholesterol particles promote inflammation, the formation of plaques and the migration of smooth muscle cells, which all contribute to the development of atherosclerotic lesions. Moreover, at the cellular level, these toxic free radicals can also damage mitochondria. Identifying chemical agents and drugs that can protect mitochondria is a rational means to prevent severe cellular damages. However, the current treatments for atherosclerosis are limited or when available adverse drug reactions occur in some patients. Previous studies on H_2_S, have found it to be a vasodilator in the cardiovascular system, yet few studies have determined the effects of H_2_S on the endothelium nor the mechanisms by which it can protect mitochondrial function and suppressing oxidative damage. To test our hypothesis that H_2_S has a protective effect on the endothelium against cell damage, we used HUVECs as a model system. Oxidative injury was induced by H_2_O_2_ treatment and the protective effects of H_2_S assessed using the donor compound, NaHS. Considering the important roles of mitochondria in cell energy production, free radical generation and cell survival, this research unveils for the first time the mechanisms involved in mitochondria protection by H_2_S in endothelial cells.

We first evaluated the toxicity of H_2_S in HUVECs. Cell viability studies indicated that H_2_S was non-toxic at the μM levels. In addition, we also demonstrated a concentration dependent protective effect of H_2_S against H_2_O_2_ induced loss of cell viability. Moreover, the protective effect of H_2_S against H_2_O_2_ may be regulated by inhibition of early apoptosis. Interestingly, H_2_S is synthesized by the enzymes CSE and cystathionine β-synthase (CBS) [12]. CSE is expressed in the cardiovascular system, liver, kidney, stomach and uterus; while CBS is chiefly located in the nervous system, liver, kidney, placenta and pancreatic islets [13]. In our study, we found that CSE gene and protein expression levels were suppressed by H_2_O_2_ treatment. This observation has been further confirmed by the noted reduction in H_2_S levels in cell lysates following H_2_O_2_ treatment. Parallel studies also found that CBS gene and protein expression levels were not significantly influenced by H_2_O_2_, H_2_S or PAG treatments. The trends in H_2_S levels, CSE and CBS gene and protein expressions correspond well with our investigations on the changes of mitochondria functions and redox status. Consequently, our observations correspond with previous findings about the localization of H_2_S synthesis enzymes, suggesting the mitochondria protective effects by exogenous H_2_S are probably though CSE/H_2_S pathway.

We further explored the mechanisms of action of H_2_S on H_2_O_2_ induced cells viability. Since mitochondrial function is linked to cell redox status, and that H_2_S is a known redox active molecule, we conducted a series of studies on mitochondria. The primary function of mitochondria is to generate ATP by the process of oxidative phosphorylation [14]. ATP synthesis is driven via the transfer of electrons through complex I to V generating a concentration gradient of protons across the inner mitochondrial membrane thus maintaining membrane potential [15]. During stress induction electron transportation and ATP synthesis often fails leading to the accumulation of free radicals and mitochondrial dysfunction [16]. In our study, ATP synthesis was increased following pre-treatment with H_2_S. In contrast, H_2_O_2_ abolished ATP synthesis. This result suggests that exogenous H_2_S stimulates efficient oxidative phosphorylation rates thus raising mitochondrial energy metabolism. This leads us to speculate that one of the mechanisms of H_2_S protection against oxidants may be directly through improved rates of ATP synthesis.

An additional factor involved in mitochondrial functions is the integrity of the mitochondrial membrane structure, which is responsible for the transmembrane proton gradient and ATP energy production [17]. During a severe stress insult, mitochondrial membrane depolarization occurs leading to organelle swelling resulting in ROS production, ATP hydrolysis and apoptosis [18]. Several lines of evidence have demonstrated that atherosclerotic lesions are promoted by mitochondrial depolarization [19] and cytochrome c redistribution [20]. Under our experimental conditions, preconditioning with NaHS attenuated the discontinuity of the outer mitochondrial membrane, stabilized mitochondrial membrane permeabilization and controlled cytochrome c release from the mitochondria to the cytosol. According to our study, H_2_S significantly inhibited cytochrome c release and preserved endothelial ultrastructure. One possible mechanism may be directly through the preservation of mitochondrial inner and outer membranes, like the cristae, and indirectly through the inhibition of matrix remodeling. Our results also showed that mitochondria dysfunction occurred as early as 4 hours following H_2_O_2_ treatment, and stimulated an increased production of ROS [10]. Therefore, protective intervention in these early stages may contribute to the amelioration of atherosclerosis damage.

The third and most important factor that should be highlighted is the interplay between mitochondrial ROS production and antioxidants. Although oxidation reactions are crucial for physiological functions, elevated levels of ROS can be damaging and toxic [21]. It has been widely acknowledged that ROS are involved in the initiation and progression stages of atherogenesis [22]. We observed that H_2_O_2_ (600 μM) strongly reduced endothelial cell viability however, pretreatment with H_2_S (300 μM NaHS), preserved cell viability. This finding correlated with a significant decrease in MDA, DPPP and endogenous ROS. Interestingly, the protective effects of H_2_S could be reversed by PAG. One possible explanation for this observation is that H_2_S is a strong reducing agent and may readily react with labile molecules, particularly those derived from reactive oxygen and nitrogen species, like the superoxide radical anion [23], hydrogen peroxide [24], peroxynitrite [25] and hypochlorite [26]. All these compounds are highly reactive and their reactions with H_2_S may contribute to the protection of mitochondria in endothelial cells. Moreover, our results also found that pretreatment with NaHS elevated the protein expressions and activities of the antioxidant enzymes SOD, catalase and glutathione peroxidase. These findings further support an antioxidant role for H_2_S. Our observations agree with recent studies that found H_2_S was cardioprotective by virtue of its antioxidant properties [27]. In this case, our results suggest that the third underlying mechanism of mitochondrial protective by exogenous H_2_S is though decreasing the toxicity of ROS via increasing the expression levels of antioxidants enzymes.

**Figure 6:**
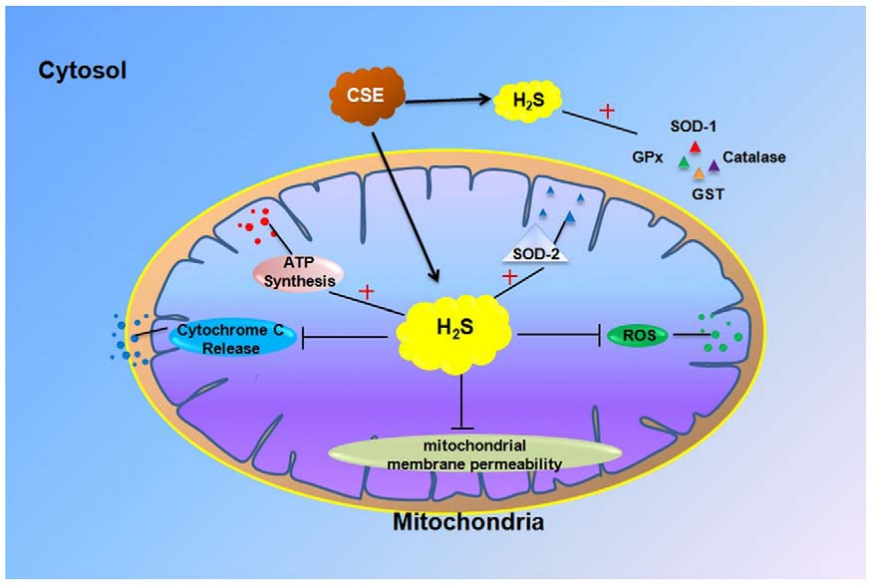
Conceptualization of the way in which H_2_S may influence on H_2_O_2_-induced cell damage by preserving mitochondrial functions and displaying antioxidative abilities though CSE/H_2_S pathway.

In conclusion, this study describes the cellular protective effects of H_2_S against H_2_O_2_-induced toxicity, as shown in Fig. 6. This protection was associated with the preservation of mitochondria function and by stimulating cellular antioxidant defenses. Collectively, the ability of the CSE/H_2_S pathway to alter oxidative conditions suggests that the modulation of CSE expression and H_2_S production may provide a novel therapeutic avenue for the treatment of atherosclerosis. This is the first attempt to link H_2_S treatment with mitochondria protection in endothelial cells and provides a new insight into the cellular protective mechanisms of H_2_S. The availability of H_2_S donors should facilitate further studies on its cardiovascular protective roles in tissue-, animal- and patient-specific studies.

## Materials and Methods

### Materials

Cysteine, PAG, DHE and LDH assay kit were purchased from Sigma, USA. NaHS was purchased from Aldrich, USA. H_2_DCFDA and Annexin V/PI kit were purchased from Invitrogen, USA. MDA levels kit, JC-1 assay kit were purchased from Beyotime, China. DPPP, kits for antioxidant enzyme assays, SOD, catalase, GPx and GST assay were purchased from Cayman Chemicals, USA.

### Cell culture

HUVECs (Lonza, Singapore) were grown in EGM-2 (Lonza, Singapore) containing vascular endothelial growth factor (VEGF), basic fibroblast growth factor (bFGF), insulin-like growth factor-1 (IGF-1), epidermal growth factor (EGF), hydrocortisone, heparin, gentamicin sulfate amphotericin, 1‰ ascorbic acid and 2% fetal bovine serum (FBS) at 37°C in a humidified atmosphere with 5% CO_2_. HUVECs were passaged every three days. The 4^th^ to 8^th^ passages of HUVECs were used for this study.

### Treatment protocols

For all experiments, HUVECs were grown to confluence in 96-well plates, 35 mm^2^ dishes or 100 mm^2^ dishes. Cells were pre-incubated with NaHS (10, 30, 100, 300, 500 or only 300 µM) or PAG (10 mM) for 6 hours before exposure to H_2_O_2_ (600 μM). Following exposure to H_2_O_2_, cells were harvested for further analysis.

### Cytotoxicity assays

The cell viability was determined by the colorimetric MTT assay. Briefly, HUVECs were seeded on 96-well plates in culture medium and maintained in regular growth medium for one day. Cells were pre-treated with different concentrations of NaHS (30, 100, 300 µM) and PAG (10 mM) for 6 hours then exposed to 600 μM H_2_O_2_ for 4 hours. Following H_2_O_2_ treatment, 10 µL of MTT (final concentration 0.5 mg/mL) was added to each well and cultures were incubated for 4 h at 37°C. The medium was then removed and the cells were washed twice with phosphate-buffered saline (PBS). The metabolized MTT was solubilized with dimethylsulfoxide and the absorbance of the solubilized blue formazin dye was read at 530 nm, with 690 nm as reference. The reduction in optical density produced by NaHS treatment was considered to represent the decrease in cell viability. The cells incubated with control medium were considered to be 100% viable. Cell viability%  =  absorbance of each injured group/absorbance of normal group ×100. The effective concentration of H_2_O_2_ and NaHS chosen for further experiments was based on these MTT results.

Cell death was determined by measuring LDH activity. At the end of incubation, the supernatant was collected, and the amount of LDH released from cells was determined using LDH assay kit according to the manufacturer's instructions. The absorbance was measured on a microplate reader at 490 nm. The data in each treatment group is expressed as a percentage of control.

### Fluorescent staining of nuclei

HUVECs nuclei were stained with chromatin dye (Hoechst 33258). Briefly, cells were fixed with 3.7% paraformaldehyde for 10 min at room temperature, washed twice with phosphate buffered solution (PBS), and incubated with 10 µM Hoechst 33258 in PBS at room temperature for 30 min. After three washes, cells were observed under a fluorescence microscope (Olympus DP72).

### Cell apoptosis assay

HUVECs were cultured in 35 mm^2^ disks. After treatments, cells were collected by trypsinization and centrifugation at 1500 rpm for 5 minutes, followed by washing cell pellet twice with cold PBS and resuspending cell pellet in 1* Annexin-binding buffer. Then, cells were added 5 µL Annexin V and 1 µL 100 µg/ml PI working solution in 100 µL cell suspension, and incubated at room temperature for 15 minutes in the dark. After the incubation, 400 µL 1* Annexin-binding buffer was added. After mixing gently, the fluorescence intensity was detected with a flow cytometry (CyAn ADP, Beckman Coulter, USA) at emission at 530 nm and 575 nm and excitation at 488 nm. The percentage of cells stained by Annexin V/PI which indicates early apoptosis was shown in bar chart.

### Measurement of H_2_S concentrations

500 μl medium was mixed with 250 μl of zinc acetate (1% w/v) in duplicates. Subsequently, NNDPD (20 μM; 133 μl) in 7.2 mol/L HCl was added, followed by FeCl_3_ (30 μM; 133 μl) in 1.2 mol/L HCl. Thereafter, TCA (10% w/v; 250 μl) was used to precipitate any protein. This final solution was then centrifuged at 24000 g for 5 min at 4°C. The optical absorbance of the resulting solution was measured at 670 nm using a 96-well microplate reader (Tecan Systems Inc., Switzerland). H_2_S concentration for each sample was calculated against a calibration curve made using NaHS standard (3.125 μM–250 μM). Results are expressed as μM.

### Measurement of CSE activity

Briefly, HUVECs were harvested by a cell lysis buffer, and then centrifuged at 24000 g for 5 min at 4°C. The supernatant was used for this assay. The reaction mixture contained 20 μl of 10 mM L-cysteine, 20 μl of 2 mM pyridoxal-5-phosphate, 30 μl of saline and 430 μl cell lysis supernatant. The catalytic reaction was initiated by transferring the reaction mixture contained in microtubes from ice to a 37°C water bath for 30 min. Then 250 μl of 1% zinc acetate was added to the tubes using a syringe to trap any evolved H_2_S. 250 μl of 10% trichloroacetic acid (TCA) was added next to quench the enzymatic reaction. Finally, 133 μl N, N-dimethyl-pphenylenediamine sulphate (NNDPD) in 7.2 M HCl and 133 μl of FeCl_3_ in 1.2 M HCl were added. The absorbance of the final reaction mixture was measured at 670 nm using a 96-well microplate reader (Tecan Systems Inc., Switzerland). All samples were assayed in duplicates. H_2_S concentration for each sample was calculated against a calibration curve made using NaHS standard (3.125 μM–250 μM). Results are expressed as μmol/h/g protein. Protein content was determined using a BCA assay kit (BIO-RAD).

### Preparation of HUVECs Mitochondria

Treated and untreated HUVECs were harvested by centrifugation at 1000 g for 3 min at room temperature. Mitochondrial and cytosol extractions were carried out using a Mitochondrial Isolation Kit (Pierce Chemical) according to manufacturer's instructions.

### ATP Synthesis Recording

1 mg/ml of mitochondria were collected for ATP synthesis analysis. The rate of ATP production was measured using a bioluminescence assay kit (Beyotime, China). Briefly, isolated mitochondria were immediately incubated with 2.5 mM ADP, 1 mM pyruvic acid and 1 mM malic acid. The ATP synthesis was kinetically recorded every 30 s for 2 minutes. Then, lyciferin substrate and luciferase enzyme were added and bioluminescence was measured by Luminometer (Varioskan Flash Multimode Reader, Thermo). Standardization was performed with known quantities of standard ATP provided in the kit and measured in the same conditions. The rate of ATP synthesis was calculated using a linear regression. Results were expressed in µmol ATP/min/g of mitochondrial protein.

### JC-1 staining

Loss of mitochondrial membrane potential (Δ*Ψ*_m_) was assessed by fluorescence microscopy using the dye 5,5′,6,6′-tetrachloro-1,1′,3,3′- tetraethylbenzimidazole- carbocyanide iodine (JC-1assay kit, Beyotime, China). After each treatment, HUVECs were stained with JC-1 for 20 min at 37°C. Cells on 8-chamber slides were scanned with a fluorescence microscope (Olympus DP72). Fluorescence was analyzed with a Texas red-FITC filter cube. Red emission of the dye represented a potential-dependent aggregation in the mitochondria, reflecting Δ*Ψ*_m_. Green fluorescence represented the monomeric form of JC-1, appearing in the cytosol after mitochondrial membrane depolarization. Cells treated with 10 µM CCCP were used as positive control. CCCP is a protonophore which can cause dissipation of ΔΨ_m_.

### Δ*Ψ*_m_ measurement

JC-1 was used to measure Δ*Ψ*_m_ of HUVECs. Total cells were collected into 2 ml tubes and incubated with JC-1 for 20 min at 37°C. The fluorescence intensity was detected with a flow cytometry (BD FACSAria I cell sorter, Becton Dickinson Company). The wavelengths of excitation and emission were 514 nm and 529 nm for detection of monomeric form of JC-1. 585 nm and 590 nm were used to detect aggregation of JC-1. The ratio of aggregated JC-1 and monomeric JC-1 represented Δ*Ψ*_m_ of HUVECs.

### Measurement of ROS

The fluorescent probe, H_2_DCFDA, was used to measure the intracellular generation of ROS by H_2_O_2_. Briefly, confluent HUVECs in 96-well plates were pretreated with 300 μM NaHS or 10 mM PAG for 6 hours and then stimulated with 600 μM H_2_O_2_ for 4 h. The reactions were stopped by removing medium, and washing with PBS followed by staining with 10 μM H_2_DCFDA for 20 min at 37°C. DHE was also used to detect ROS production. After drug treatments, cells were incubated in 5 μM DHE for 30 min at 37°C. The fluorescence intensities of H_2_DCFDA and DHE were kinetically measured at an excitation and emission wavelength of 485 nm and 530 nm for H_2_DCFDA, and 520 nm and 610 nm for DHE, respectively, using a fluorescent microplate reader (Molecular Devices, Gemini XS, USA).

### Lipid peroxidation assays

HUVECs were cultured in 35 mm^2^ disks. A cell lysis buffer (RIPA buffer) was used to the collect cell samples. MDA levels were determined by measuring the thiobarbituric acid-reactive substances using a commercial kit (Beyotime, China) according to the manufacturer's introduction. MDA values are expressed as nmol/mg protein.

Lipid peroxidation was further estimated using a fluorescent probe, DPPP. After treatments, cells were incubated with 100 μM DPPP for 60 min in the dark. The fluorescent intensities of DPPP fluorescence were analyzed with a fluorescent microplate reader (Molecular Devices, Gemini XS, USA) at an excitation of 351 nm and an emission of 380 nm.

### Cytochrome c Release Assay

Cells were harvested by centrifugation at 1000 g for 3 min at room temperature. Mitochondrial and cytosol extractions were separated using the Mitochondrial Isolation Kit (Pierce Chemical) according to manufacturer's instructions. The presence of cytochrome c was detected from mitochondrial and cytosol extractions by immunoblot analysis using anti-cytochrome c antibody (1∶1000, Cell Signaling).

### Transmission Electron Microscopy

Cells were harvested then prefixed in 2.5% glutaraldehyde solution overnight. Postfixation was in cold 1% aqueous osmium tetroxide for 1 h. After rinsing with PBS, the samples were dehydrated in a graded ethanol series of 25 to 100% and then embedded in fresh resin and polymerized at 60°C for 24 h. Ultra-thin sections were sliced with glass knives on a LKB-V ultramicrotome (Leica), stained with uranyl acetate and lead citrate, and examined under a transmission electron microscope CM120 Bio TWIN (Philips).

### Antioxidant enzyme activities assay

HUVECs were cultured in 100 mm^2^ disks. Cells were harvested through centrifuging (1,500 g for 10 minutes at 4°C). Then, cells were homogenized in cold buffer and centrifuged at 10,000 g for 15 min at 4°C. The supernatants were used for all the assays. The antioxidants assays were performed using commercially available kits.

### Immunoblotting

Cultured HUVECs were harvested by scraping and centrifugation, washed twice with ice-cold PBS, and re-suspended in RIPA buffer. Soluble proteins were collected by centrifugation at 15,000 g for 15 min. Protein lysates were subjected to 10–12% SDS-PAGE and transferred onto a PVDF membrane (Millipore Corporation). After blocking with 5% skim milk, the membranes were incubated with the respective primary antibodies (SOD-1 1∶800, SOD-2 1∶400, catalase 1∶500, GPx 1∶500, GST 1∶500, cytochrome c 1∶1000, CTH 1∶1000, Santa Cruz; CBS 1∶800 Abcam) in PBS 0.1% Tween-20 overnight at 4°C. The membranes were then incubated with the appropriate secondary horseradish peroxidase-conjugated IgG antibodies at a 1∶10,000 dilution for 1 h at room temperature (Santa Cruz). Immunoreactive proteins were then visualized enhanced chemoluminescence (Pierce). The signals were quantified by densitometry using a Kodak Image Station 4000R (Kodak). β-actin served as the loading control. Protein content was determined using a BCA assay kit (BIO-RAD).

### Real-time PCR

Total RNA from HUVECs was isolated using Trizol reagent (Invitrogen, Carlsbad, CA). Real-time PCR was performed in triplicate on a Corbett RG6000 5plex with HRM sequence detector, using 100 ng RNA, 0.1 μl PCR master Mix (QuantiTect, QIAGEN), 5 μl SYBR (QuantiTect, QIAGEN), and 5 μM each of forward and reverse primers, in a final volume of 10 μl. Samples were incubated at 50°C for 30 min, then at 95°C for 15 min; denaturation was performed for 45 cycles at 94°C for 15 s, and followed by annealing and extension at 62°C for 30 s and 72°C for 30 s. Amplifications were normalized by β-actin. The amount of the target gene, normalized to β-actin, is given as 2^−ΔΔCT^. Results were analyzed using the Rotor-Gene series software (1.7). Expressions of CSE and CBS mRNA were determined.

The primers used for real-time PCR are followed: CSE 5′-CCATCTCCTATTGATTGTTACCTCT-3′ and 5′-CACTGACGCTTCACCAACTC-3′; CBS 5′-TCAAGAGCAACGATGAGGAG-3′ and 5′-ATGTAGTTCCGCACTGAGTC-3′; β-actin 5′-GAGAGGGAAATCGTGCGTGAC-3′ and 5′-CTGCTGGAAGGTGGACAGTGAG-3′.

### Statistical analysis

All values are represented as means ± SEM. One-way analysis of variance (ANOVA) was used to determine statistical significance between groups and also the two-tailed Student's t-test. A Chi-square test was employed for calculating the significance of mortality data. A probability value of <0.05 was taken to indicate statistical significance.
